# Educational value of pocket-sized ultrasound devices to improve understanding of ultrasound examination principles and sonographic anatomy for medical student

**DOI:** 10.1371/journal.pone.0185031

**Published:** 2017-09-29

**Authors:** Eun Young Kim, Kwi Hwa Park, Seung Joon Choi, Wook-Jin Chung

**Affiliations:** 1 Department of Radiology, Gachon University Gil Medical Center, Incheon, Republic of Korea; 2 Department of Medical Education, Gachon University College of Medicine, Incheon, Republic of Korea; 3 Division of Cardiology, Heart Center, Gachon University Gil Medical Center, Incheon, Republic of Korea; Waseda University, JAPAN

## Abstract

**Purpose:**

Medical students must understand the principles of ultrasonography (US), because US examinations are an important component of patient care in clinical practice. Pocket-sized ultrasound devices have the benefits of accessibility and ease of use. The primary objective of the present study was to evaluate the educational value of these devices in terms of improving medical student interest and understanding of US and sonographic anatomy.

**Methods:**

We added a US training program comprised of a self-study learning module and a hands-on training session to a two-week block curriculum of medical imaging for first year medical students (n = 40). Multiple pocket-sized US devices were used on a small-group basis during a single afternoon. Students were asked to complete a questionnaire before and after the US training session; these two questionnaires contained 6 and 10 questions, respectively, which were rated by students using a five-point Likert scale. In addition, understanding of sonographic anatomy was tested before and after the training program.

**Results:**

Forty students completed the two questionnaires and the anatomy-related tests. Students found the program educationally valuable (4.37 ± 0.54 of 5) and reported that US practice was useful for improving their understanding of the principles of US examinations (4.23 ± 0.66 of 5) and sonographic anatomy (4.40 ± 0.55 of 5). Overall confidence at performing US examinations and understanding of sonographic anatomy were significantly increased after US training (increased overall confidence score, 1.87 ± 0.91 and improvement in sonographic anatomy score, 6.55 ± 1.55, *p* values < 0.001).

**Conclusion:**

US training using pocket-sized ultrasound devices was found to be educationally valuable for medical students in terms of improving understanding of US principles and familiarizing students with sonographic anatomy.

## Introduction

Ultrasound (US) is a safe and non-invasive imaging modality that is commonly used in health care, and familiarity with this modality is important for medical students in terms of future clinical practice. Technical advances have reduced the sizes and increased the portability of US equipment. Bedside US performed using a portable device plays a critical role in point-of-care because it provides important information on, for example, volume status and the presence of fluid collection or pneumothorax, which greatly affect treatment [1].

Given increasing recognition of the value and availability of US, US training for undergraduate medical students is progressively being implemented in medical schools. Furthermore, several studies have shown US training can improve the performance and reliability of physical examinations and facilitate understanding of anatomy [2]. In addition, recently developed portable, pocket-sized US devices have obvious benefits for the education of medical students because of their portability and ease of use. Accordingly, the objective of this study was to evaluate the educational value of a pocket-sized US device in terms of familiarizing medical students with the US technique and sonographic anatomy.

## Materials and methods

### Medical students

Ethics approval for this study was granted by the Gil Medical Center Institutional Review Board (approval number: GAIRB-2016-079). We recruited first-year students (n = 40) attending a regular course of a two-week block curriculum on medical imaging at Gachon University College of Medicine (Incheon, South Korea). All 40 students volunteered to participate in the study and provided written informed consent. This two-week block curriculum was the first block of the clinical medicine curriculum. Course teachers were radiologists, and the goals of this block curriculum were to provide understanding of the principles of modern medical imaging modalities (radiography, US, computed tomography, and magnetic resonance imaging), their proper utilization and appropriateness, and to enable students to interpret anatomic images.

### US equipment

**Fig 1:**
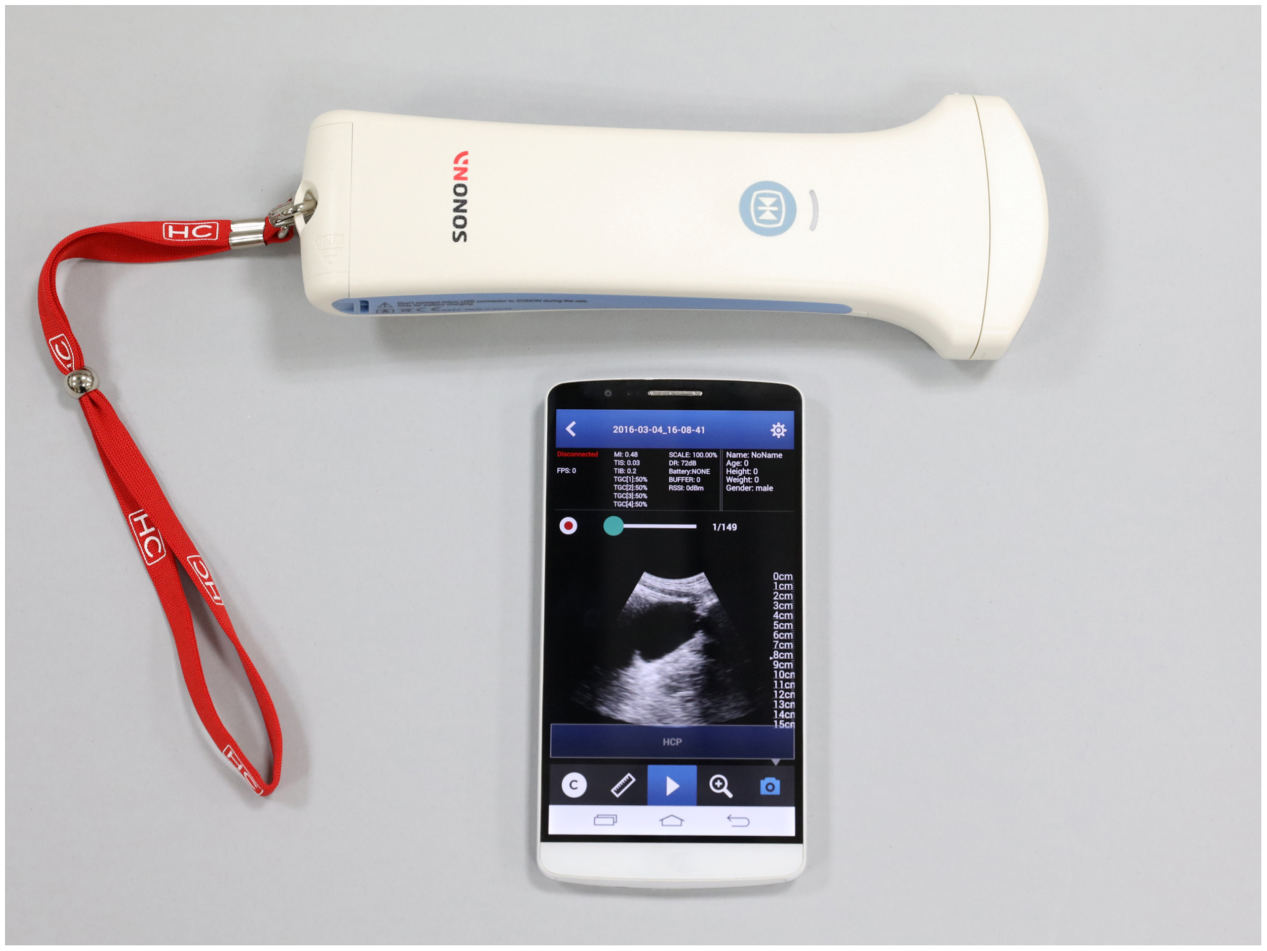
Photograph of the pocket-sized ultrasound device. Images were displayed on a smartphone via a device-generated wireless (free Wi-Fi) connection. Under a Creative Commons (CC) license permission to publish this picture was obtained from HEALCERION, Seoul, Korea (S2 File).

US training was performed using the SONON 300C portable pocket-sized US device (HEALCERION, Seoul, Korea). The US probe and associated electronics were combined in a single unit, and images were visualized and recorded on a smartphone or tablet via a device-generated wireless (free Wi-Fi) connection. The device measures 219 × 78 × 38 mm and weighs 390 g when fitted with a battery and a convex probe (3.5 MHz) (Fig 1). It is rechargeable and provides two-dimensional greyscale images at imaging depths of 5 to 20 cm. A total of ten units of US device were purchased by Gachon University College of Medicine in 2014 specifically for educational purposes.

### US training session

Our US training goals for first-year-medical students were as follows: 1) to increase interest in US examinations; 2) to improve basic understanding of the principles of US imaging; and 3) to identify normal abdominal structures and Morison’s pouch, which are US fundamentals in many subspecialties.

Due to limitations in time and teaching resources, US training was conducted in two sessions, as previously described [3], that is, by self-study using an independent learning module (ILM) and in a single small-group hands-on training session.

During the first session, we used a “flipped classroom model” [4] to maximize the efficiency of face-to-face teaching. This introductory ILM was created in PowerPoint to address basic issues. The module was composed of 40 slides, which provided objectives and an overview of the class (4 slides), basic physics of US (15 slides), the advantages and limitations of US examinations (5 slides), orientations of transducers and the sonographic findings of normal abdominal organs, and the location of Morison’s pouch (16 slides). The presentation was created by a teaching radiologist familiar with course contents and the medical school curriculum. After thirty minutes of self-review by students, an overall review was performed by the same radiologist to ensure that the module contents were appropriate for first-year medical students. Students also have the opportunity to interact with the radiologist and ask questions.

During the second session, a radiologist explained how to operate the pocket-sized US, provided scanning tips, and performed a trial US examination on a volunteer student’s abdomen in the classroom. Due to the small sizes of smartphone screens, the demonstration was viewed on a projector screen. Thereafter, hands-on US training was performed in multiple small-group settings to ensure that each student had adequate opportunity to practice scanning. For this purpose, the 40 students were divided into eight groups of five. One radiologist, two second-year radiology residents, and two US sonographers were recruited for the hands-on session. These eight groups were assigned to individual rooms, and each group was assigned a pocket-sized US. The members of each group scanned other members that volunteered to be scanned, which gave all participants the opportunity to practice. Four instructors oversaw activities in two rooms each, and one instructor kept the program under close observation. The students had the opportunity to interact with instructors and to ask questions during US practice.

### Assessment

Before the first session, students were asked to voluntarily complete a questionnaire that addressed prior experience and general perceptions of US examination and baseline knowledge of US imaging principles and sonographic anatomy (Table 1, S1 File). After the second session had been completed, students were asked to complete a second questionnaire that requested their opinions of the US training session (Table 2). Responses to questions were rated using a five-point Likert scale. In addition, ten questions on sonographic anatomy were put to students before and after US training (Table 3). About four hours elapsed between the pre- and post-training testing. Discussion between students was prohibited during the assessment.

**Table 1:** Survey questions, answer choices, and responses from first-year medical students before ultrasound (US) training.

Survey question	Answer choice	Number of responses, n (%)	Mean response (SD)
I have used US prior to medical school.	5 Strongly agree	1 (2.5)	1.40 (0.87)
	4 Agree	0	
	3 Neutral	4 (10.0)	
	2 Disagree	4 (10.0)	
	1 Strongly disagree	31 (77.5)	
Understanding US principles is difficult for me.	5 Strongly agree	3 (7.5)	3.25 (0.87)
	4 Agree	12 (30.0)	
	3 Neutral	17 (42.5)	
	2 Disagree	8 (20.0)	
	1 Strongly disagree	0	
I can explain how US can make images.	5 Strongly agree	0	1.67 (0.66)
	4 Agree	0	
	3 Neutral	4 (10.0)	
	2 Disagree	19 (47.5)	
	1 Strongly disagree	17 (42.5)	
I am keen to practice US.	5 Strongly agree	15 (37.5)	4.22 (0.70)
	4 Agree	19 (47.5)	
	3 Neutral	6 (15.0)	
	2 Disagree	0	
	1 Strongly disagree	0	
I expect that US training can facilitate US principles.	5 Strongly agree	17 (42.5)	4.27 (0.75)
	4 Agree	18 (45.0)	
	3 Neutral	4 (10.0)	
	2 Disagree	1 (2.5)	
	1 Strongly disagree	0	
I expect that US training can facilitate the learning of sonographic anatomy.	5 Strongly agree	13 (32.5)	4.10 (0.78)
	4 Agree	19 (47.5)	
	3 Neutral	7 (17.5)	
	2 Disagree	1 (2.5)	
	1 Strongly disagree	0	

**Table 2:** Survey questions, answer choices, and responses from first-year medical students after ultrasound (US) training.

Survey Question	Answer choice	Number of responses, n (%)	Mean response (SD)
Overall, this US training was educationally valuable.	5 Strongly agree	16 (40.0)	4.37 (0.54)
	4 Agree	23 (57.5)	
	3 Neutral	1 (2.5)	
	2 Disagree	0	
	1 Strongly disagree	0	
Overall, this program increased my interest in radiology.	5 Strongly agree	13 (32.5)	4.25 (0.59)
	4 Agree	24 (60.0)	
	3 Neutral	3 (7.5)	
	2 Disagree	0	
	1 Strongly disagree	0	
This program improved my understanding of US principles.	5 Strongly agree	14 (35.0)	4.23 (0.66)
	4 Agree	21 (52.5)	
	3 Neutral	5 (12.5)	
	2 Disagree	0	
	1 Strongly disagree	0	
This program improved my understanding of sonographic anatomy.	5 Strongly agree	17 (42.5)	4.40 (0.55)
	4 Agree	22 (55.0)	
	3 Neutral	1 (2.5)	
	2 Disagree	0	
	1 Strongly disagree	0	
The time (30 minutes) given for the introductory module was adequate for understanding.	5 Strongly agree	9 (22.5)	3.75 (1.03)
	4 Agree	20 (50.0)	
	3 Neutral	3 (7.5)	
	2 Disagree	8 (20.0)	
	1 Strongly disagree	0	
The introductory module was helpful for hands-on training.	5 Strongly agree	18 (45.0)	4.43 (0.55)
	4 Agree	21 (52.5)	
	3 Neutral	1 (2.5)	
	2 Disagree	0	
	1 Strongly disagree	0	
The in-person instruction during hands-on was educationally valuable.	5 Strongly agree	20 (50.0)	4.47 (0.55)
	4 Agree	19 (47.5)	
	3 Neutral	1 (2.5)	
	2 Disagree	0	
	1 Strongly disagree	0	
Small groups (5 students in a group) were adequate for hands-on practice.	5 Strongly agree	16 (40.0)	4.27 (0.72)
	4 Agree	20 (50.0)	
	3 Neutral	3 (7.5)	
	2 Disagree	1 (2.5)	
	1 Strongly disagree	0	
The US training was at an appropriate level to teach first-year students.	5 Strongly agree	8 (20.0)	3.85 (0.86)
	4 Agree	21 (52.5)	
	3 Neutral	9 (22.5)	
	2 Disagree	1 (2.5)	
	1 Strongly disagree	1 (2.5)	
Hand-held ultrasonography was convenient for practice.	5 Strongly agree	15 (37.5)	4.28 (0.64)
	4 Agree	21 (52.5)	
	3 Neutral	4 (10.0)	
	2 Disagree	0	
	1 Strongly disagree	0	

**Table 3:** Results of the survey and ten questions that addressed confidence to perform an ultrasound (US) examination and correct localization of sonographic images before and after US training.

	Before US training	After US training	Increased confidence score after US training	*p* value
I can confidently explain US principles (#1).	2.38 (0.84)	4.00 (0.60)	1.63 (0.95)	< .001^†^
I can confidently localize abdominal organs on US (#2).	1.68 (0.66)	3.53 (0.64)	1.85 (0.86)	< .001^†^
I can confidently localize Morison’s pouch (#3).	1.48 (0.64)	3.63 (0.67)	2.15 (0.86)	< .001^†^
Overall confidence (#1–3)	1.84 (0.81)	3.72 (0.66)	1.87 (0.91)	< .001^†^
Scores of ten questions about sonographic anatomy	2.07 (1.25)	8.62 (1.13)	6.55 (1.55)	< .001^†^

Note—Values are means (standard deviations),

^†^Paired T-test

### Statistical analysis

Descriptive statistics are reported as absolute numbers and percentages of students. Likert scale answers were summarized using mean values and standard deviations (Tables 1 and 2). The paired t-test was used to determine the significances of differences between responses to questions asked pre- and post-US training. *p* values < 0.05 were considered statistically significant, and the analysis was performed using SPSS ver. 19.0 for Windows (SPSS Inc., Chicago, IL).

## Results

All students (n = 40) completed the self-study and hands-on US training sessions, and all students completed the questionnaires and answered the questions before and after US training sessions.

When surveyed prior to US training, most students reported little (2.5%) exposure to US prior to medical school. The majority (90.0%) of students were unaware of how images are produced using ultrasound. On the other hand, 85.0% were “interested” in learning more about US (Table 1).

After completing US training, almost all students (97.5%) found the training to be educationally valuable (4.37 ± 0.54 of 5) and that the program improved their understanding of US imaging principles (4.23 ± 0.66 of 5) and sonographic anatomy (4.40 ± 0.55 of 5). The introductory ILM was found to be helpful for hands-on training (4.43 ± 0.55 of 5), and the students found the training appropriate for first-year-medical students (3.85 ± 0.86 of 5) (Table 2).

In addition, overall confidence regarding understanding the principles of US examination and of performing US examinations increased significantly (increased overall confidence score, 1.87 ± 0.91, *p* <0.01), and mean sonographic anatomy score was also significantly improved after US training (improved score, 6.55 ± 1.55, *p* < 0.001) (Table 3).

## Discussion

Several studies have reported that US education improves understanding of anatomy and reinforces the physical examination skills of medical students [1, 2, 5, 6], and it has been reported medical students are able to perform standard sonographic techniques reliably after US training [2, 3]. The present study also reveals the educational value of US training for medical students. In particular, US training increased preclinical student interest about US examinations, improved their basic understanding of the principles of US imaging, and enabled students to correctly locate normal abdominal structures and Morison’s pouch.

Although it is being progressively adopted, the majority of medical schools have not yet included an US training program in their formal curricula, presumably because of a lack of; time in existing curricula, available US units, level-appropriate teaching materials, and a lack of qualified teachers for hands on training. In the present study, the use of a self-study learning module and multiple pocket-sized ultrasound devices enabled time-effective and iterative small-group based training. The flipped classroom model used in the first phase of our training program made effective use of instructors’ time and maximized hands-on training time, since the self-study learning module provided students opportunities to ask questions and interact with the instructing radiologist. Furthermore, the use of multiple pocket-sized US units, which is a unique aspect of the present study, facilitated effective, iterative small-group training during a single afternoon session. The efficacy and diagnostic impact of pocket-size portable ultrasonography have been previously demonstrated by experts and non-experts [5, 7].

The concept of teaching medical students how to perform US examinations is somewhat controversial [3]. Current medical education focuses on the proper utilization and appropriateness of imaging modalities for students, because the majority of students are likely to become referring physicians. However, US examinations are currently being performed by clinicians in a wide variety of specialties for point-of-care or bedside procedural guidance or as adjuncts to physical examinations [8, 9]. Accordingly, several medical schools have incorporated hands-on US training into their formal curricula [10, 11]. It should be noted that the benefits to formal instruction include better quality control and standardization of training and the opportunity to improve competency. In the present study, preclinical medical students found US training educationally beneficial, and the surveys and tests performed revealed improved confidence at performing US examinations and ability to identify abdominal organs and Morison’s pouch.

Several limitations of the present study should be mentioned. First, the sample size was small. Second, because of its survey-based design, results are dependent on the validity of self-reported data. Although no validation study was conducted on the questionnaires used; they were adopted and modified from a previously published study [3]. In addition, we verified the educational value of the training program by testing understanding of sonographic anatomy before and after US training. Third, we did not evaluate the long-term retention of US concepts or of sonographic anatomy taught during the US training session. We suggest future studies focus on the long-term benefits of early exposure to US technology and practice at medical schools in the contexts of patient care and training.

In conclusion, US training using pocket-sized ultrasound devices was found to be educationally valuable in terms of improving understanding of US principles and of familiarizing students with sonographic anatomy.

## Supporting information

S1 File Questionnaire for students' opinions on ultrasound training. (DOCX)

S2 File Content-permission-form. (PDF)
