# Supplementary material for: Educational value of pocket-sized ultrasound devices to improve understanding of ultrasound examination principles and sonographic anatomy for medical student
Source: PLoS One. 2017 Sep 29;12(9):e0185031. doi: 10.1371/journal.pone.0185031 (PMC5621680; doi:10.1371/journal.pone.0185031)
Supplement: S1 File — (DOCX) [file pone.0185031.s001.docx]

**Pre-survey of students' opinions on ultrasound training**

■ Student ID:

※ This questionnaire is intended to be a pre-survey of students' opinions on ultrasound training. Please indicate the degree of agreement.

| Survey questions | | Strongly agree | agree | Neutral | Disagree | Strongly disagree |
| --- | --- | --- | --- | --- | --- | --- |
| 1 | I have used US prior to medical school | 5 | 4 | 3 | 2 | 1 |
| 2 | Understanding US principles is difficult for me | 5 | 4 | 3 | 2 | 1 |
| 3 | I can explain how US can make images | 5 | 4 | 3 | 2 | 1 |
| 4 | I am excited to practice US | 5 | 4 | 3 | 2 | 1 |
| 5 | I expect that US training can facilitate US principles | 5 | 4 | 3 | 2 | 1 |
| 6 | I expect that US training can facilitate the learning of sonographic anatomy | 5 | 4 | 3 | 2 | 1 |
| 7 | I can confidently explain US principles | 5 | 4 | 3 | 2 | 1 |
| 8 | I can confidently localize abdominal organs on US | 5 | 4 | 3 | 2 | 1 |
| 9 | I can confidently localize Morison’s pouch | 5 | 4 | 3 | 2 | 1 |

**Post-survey of students' opinions on ultrasound training**

■ Student ID:

※ This questionnaire is intended to be a post-survey of students' opinions on ultrasound training session. Please indicate the degree of agreement.

| Survey questions | | Strongly agree | agree | Neutral | Disagree | Strongly disagree |
| --- | --- | --- | --- | --- | --- | --- |
| 1 | Overall, this US training was educationally valuable | 5 | 4 | 3 | 2 | 1 |
| 2 | Overall, this program increased my interest in radiology | 5 | 4 | 3 | 2 | 1 |
| 3 | This program improved my understanding of US principles | 5 | 4 | 3 | 2 | 1 |
| 4 | This program improved my understanding of sonographic anatomy | 5 | 4 | 3 | 2 | 1 |
| 5 | The time (30 minutes) given for the introductory module was adequate for understanding | 5 | 4 | 3 | 2 | 1 |
| 6 | The introductory module was helpful for hands-on training | 5 | 4 | 3 | 2 | 1 |
| 7 | The in-person instruction during hands-on was educationally valuable | 5 | 4 | 3 | 2 | 1 |
| 8 | Small groups (3-4 students in a group) were adequate for hands-on practice | 5 | 4 | 3 | 2 | 1 |
| 9 | The US training was at an appropriate level to teach first-year students | 5 | 4 | 3 | 2 | 1 |
| 10 | Hand-held ultrasonography was convenient for practice | 5 | 4 | 3 | 2 | 1 |
| 11 | I can confidently explain US principles | 5 | 4 | 3 | 2 | 1 |
| 12 | I can confidently localize abdominal organs on US | 5 | 4 | 3 | 2 | 1 |
| 13 | I can confidently localize Morison’s pouch | 5 | 4 | 3 | 2 | 1 |
