# Supplementary material for: Educational value of pocket-sized ultrasound devices to improve understanding of ultrasound examination principles and sonographic anatomy for medical student
Source: PLoS One. 2017 Sep 29;12(9):e0185031. doi: 10.1371/journal.pone.0185031 (PMC5621680; doi:10.1371/journal.pone.0185031)
Supplement: S2 File — (PDF) [file pone.0185031.s002.pdf]

Request for Permission to Publish Content under CC-BY License

Dear Rights Holder or Representative,

I have submitted a paper for publication in a PLOS journal, and wish to include the content listed below in the paper. I'm hereby requesting your (or your company's or institution's) permission to include the content in my paper. Please note that all PLOS journals are published under a Creative Commons Attribution License (CC BY), which allows for unrestricted use and distribution, even commercial, as long as attribution is given to the creator or rights holder of the content. See https://creativecommons.org/licenses/by/4.0/.

To grant me permission to use the content in my PLOS paper, please fill in the information below and then scan the completed form and send it to me at my email address.

Thank you.

My name:

Kwi Hwa Park, PhD

My email address:

ghpark@gachon.ac.kr

Description of the content which I'm seeking permission to use (citation and/or title, and pasted screen shot, if applicable):

Figure 1

Link to the Content:

(attached Figure 1)

* * *

On behalf of myself or the rights holder, I hereby grant the permission sought herein.

Signature of Party Granting Permission:

Date:

Jul 21, 2017

Printed Name and Title:

Jeongwon Ryu, MD, C.E.O., Healcerion Co., Ltd, Seoul
